# The Risk of Ventricular Dysrhythmia or Sudden Death in Patients Receiving Serotonin Reuptake Inhibitors With Methadone: A Population-Based Study

**DOI:** 10.3389/fphar.2022.861953

**Published:** 2022-04-20

**Authors:** Tony Antoniou, Daniel McCormack, Mina Tadrous, David N. Juurlink, Tara Gomes

**Affiliations:** ^1^ Department of Family and Community Medicine, Unity Health Toronto, Toronto, ON, Canada; ^2^ Li Ka Shing Knowledge Institute, Unity Health Toronto, Toronto, ON, Canada; ^3^ Department of Family and Community Medicine, University of Toronto, Toronto, ON, Canada; ^4^ Institute for Clinical Evaluative Sciences, Toronto, ON, Canada; ^5^ Leslie Dan Faculty of Pharmacy, University of Toronto, Toronto, ON, Canada; ^6^ Women’s College Research Institute, Toronto, ON, Canada; ^7^ Sunnybrook Research Institute, Toronto, ON, Canada; ^8^ Department of Medicine, University of Toronto, Toronto, ON, Canada; ^9^ Institute for Health Policy, Management and Evaluation, University of Toronto, Toronto, ON, Canada

**Keywords:** methadone, serotonin reuptake inhibitor, nested case control studies, sudden cardiac arrest, pharmacoepidemiogy

## Abstract

**Background:** Methadone is associated with ventricular dysrhythmias and sudden death. Serotonin reuptake inhibitors (SRIs) may increase the risk of these events either by inhibiting metabolism of methadone’s proarrhythmic (S)-enantiomer, additive QT interval prolongation, or both. We sought to determine whether certain SRIs were associated with a higher risk of methadone-related ventricular dysrhythmias or sudden death.

**Methods:** We conducted a nested case-control study of Ontario residents receiving methadone between April 1, 1996 and December 31, 2017. Cases, defined as patients who died of sudden cardiac death or were hospitalized with a ventricular dysrhythmia while on methadone, were matched with up to four controls who also received methadone on age, sex, and a disease risk score. We determined the odds ratio (OR) and *p*-value functions for the association between methadone-related cardiotoxicity and treatment with SRIs known to inhibit metabolism of (S)-methadone (paroxetine, fluvoxamine, sertraline) or prolong the QT interval (citalopram and escitalopram). Patients who were not treated with an SRI served as the reference group.

**Results:** During the study period, we identified 626 cases and 2,299 matched controls. Following multivariable adjustment, we found that recent use of sertraline, fluvoxamine or paroxetine (adjusted OR 1.30; 95% confidence intervals [CI] 0.90–1.86) and citalopram and escitalopram (adjusted OR 1.26; 95% CI 0.97–1.63) were associated with small increases in the risk methadone-related cardiac toxicity, an assertion supported by the corresponding *p*-value functions.

**Interpretation:** Certain SRIs may be associated with a small increase in cardiac toxicity in methadone-treated patients.

## Introduction

Methadone is a long-acting opioid used primarily as treatment for opioid use disorder (Bell and Strang, 2020). However, methadone maintenance therapy can be complicated by QT interval prolongation in up to 50% of patients (Fanoe et al., 2007; Anchersen et al., 2009; Fareed et al., 2013; Chowdhury et al., 2015; Titus-Lay et al., 2021), with case reports and pharmacovigilance data describing the potential for ensuing Torsade de Pointes and sudden cardiac death (Chugh et al., 2008; Stringer et al., 2009; Kao et al., 2013; Kao et al., 2015). Risk factors for QT interval prolongation and sudden cardiac death are well described, and include older age, female sex, electrolyte abnormalities, and underlying heart disease (Chugh, 2010; Tisdale et al., 2013; Trinkley et al., 2013).

Drug interactions are another important and potentially avoidable risk factor for ventricular dysrhythmias in patients receiving methadone (Stringer et al., 2009). Because co-occurring mental health illness is common in methadone-treated patients and serotonin reuptake inhibitors (SRIs) are the most commonly prescribed class of antidepressants, the likelihood of co-prescription and potential interaction with methadone is high (Callaly et al., 2001; Rosen et al., 2008; Audi et al., 2018; Kane, 2021). However, SRIs differ in their propensity for causing drug interactions because of variable effects on drug metabolizing cytochrome P450 (CYP) isoenzymes as well as the QT interval (Hemeryck and Belpaire, 2002; Beach et al., 2014). This is especially relevant in the case of methadone, which is commercially available as a racemic mixture containing equal amounts of the (R)- and (S)-methadone enantiomers, each of which has distinct clinical and pharmacokinetic properties (Chang et al., 2011). Specifically (R)-methadone is an opioid agonist while (S)-methadone is associated with QT prolongation, increasing the risk of ventricular dysrhythmia and sudden death (Kristensen et al., 1995; Eap et al., 2007; Ansermot et al., 2010). Importantly, each enantiomer is metabolized by different CYP450 enzymes, with the CYP2B6 isoenzyme demonstrating stereoselectivity toward (S)-methadone (Chang et al., 2011; Dobrinas et al., 2013). Concomitantly administered medications that inhibit CYP2B6 may increase (S)-methadone concentrations and therefore increase the risks of dysrhythmia and sudden death. Among SRIs, prior studies have found that fluvoxamine and paroxetine increase concentrations of (S)-methadone by 30–50%, with no such increase observed with fluoxetine (Eap et al., 1997; Begré et al., 2002). In a study of 16 patients receiving methadone, sertraline, which inhibits CYP2B6, was also found to increase methadone levels by 26% (Hamilton et al., 2000). In addition to pharmacokinetic interactions, methadone-related dysrhythmia and sudden death can occur with the concurrent use of additional QT-prolonging drugs. Among SRIs, citalopram and escitalopram are associated with greater QT prolongation and a higher risk of sudden cardiac death than other agents (Castro et al., 2013; Beach et al., 2014; Assimon et al., 2019). The potential for a clinically important interaction between citalopram and methadone was highlighted by a study of forensic toxicological records in the United States, in which a strong signal for drug fatality with combined use was detected (Saad et al., 2018).

However, despite these data, the cardiac safety of combining SRIs with methadone is unknown. We sought to characterize the risk of ventricular dysrhythmias and sudden death in patients receiving these drug combinations in clinical practice. We speculated that, owing to pharmacokinetic and pharmacodynamic interactions, patients treated with methadone and either sertraline, paroxetine, fluvoxamine, citalopram or escitalopram would be at higher risk of these events relative to patients who were not prescribed SRIs.

## Materials and Methods

### Setting

We conducted a nested case-control study of Ontario residents treated with publicly funded methadone maintenance therapy between 1 April 1996 and 31 December 2017. These individuals had universal access to hospital care, physicians’ services, and prescription drug coverage.

### Data Sources

We identified prescription records using the Ontario Drug Benefit (ODB) Database, which contains comprehensive records of prescription medications dispensed to Ontario residents whose prescriptions costs are reimbursed by the provincial government. Approximately 70% of methadone-treated patients in Ontario obtain their medication through the ODB program. Methadone prescriptions are recorded in the ODB database for each date on which the drug is dispensed. We obtained hospitalization and emergency department visit data from the Canadian Institute for Health Information Discharge Abstract Database and National Ambulatory Care Reporting System, respectively. We used the Ontario Health Insurance Plan database to identify claims for physician services and used validated disease registries to define the presence of diabetes (Hux et al., 2002), hypertension (Tu et al., 2007), and congestive heart failure (Schultz et al., 2013). We obtained basic demographic data from the Registered Persons Database, a registry of all Ontario residents eligible for health insurance. We ascertained sudden death using the Ontario Registrar General Death database, which contains the cause of death reported on individual death certificates. These datasets were linked using unique encoded identifiers, analyzed at ICES, and are routinely used to study the consequences of drug interactions (Antoniou et al., 2015; Gomes et al., 2017).

### Study Subjects

We defined case patients as those who died of sudden cardiac death or were hospitalized with ventricular dysrhythmia or cardiac arrest (see Supplementary Table 1 for International Classification of Disease and Related Health Problems, ninth and 10th revision codes) on the day of or within 1 day after receiving a prescription for methadone. Previous studies evaluating the accuracy of these codes show positive predictive values exceeding 80% (De Bruin et al., 2005; Chung et al., 2010; Tamariz et al., 2012; Qirjazi et al., 2016).

We defined the index date as the date of death or hospitalization, with only the first instance of hospitalization considered for patients with more than one admission during the study period. In cases where individuals had multiple methadone claims on a given day, we assumed that the individual was exposed to methadone for the number of days corresponding to the number of claims. For example, an individual with three methadone claims on a Monday was assumed to be exposed to methadone until Wednesday and could become a case patient if they experienced sudden cardiac death or ventricular dysrhythmia on any day between Monday and Thursday (i.e., within 1 day of methadone exposure). The index date for potential controls was randomly assigned according to the distribution of index dates for included cases. For each case, we selected up to four controls from the same cohort of patients receiving methadone who were alive on their randomly selected index date. We excluded individuals with a prior diagnosis of cardiac arrest or dysrhythmia within 5 years of the index date and individuals receiving palliative care in the 6 months preceding the index date. We also excluded patients (i.e., <5 cases, 70 controls) who filled prescriptions for multiple SRIs in the 90 days preceding the index date to avoid the potential confounding effects of multiple SRI exposures. We required that all study patients have at least one methadone prescription on their index date or the day preceding it, and at least 6 months of continuous eligibility for public drug benefits prior to their index date.

To increase the comparability of cases and controls, we used a disease risk score as a confounder summary score to generate predicted probabilities of sudden cardiac death or ventricular dysrhythmia (Arbogast et al., 2008). We selected this approach because of the large number of potential confounders relative to the number of events and to attempt to balance the determinants of our outcome and baseline outcome risk among cases and controls. The disease risk score was derived for each individual using a non-parsimonious multivariable logistic regression model that included our study outcome as the dependent variable and an extensive list of demographic and clinical characteristics related to the risk of this outcome (Supplementary Material—Covariates Included in Disease Risk Score). We matched each case with up to four controls on their disease risk score (within 0.2 standard deviations), age (within 3 years), and sex. When fewer than four control subjects were available for each case, we analyzed only those controls and maintained the matching process. We excluded cases that could not be matched to at least one control.

### Exposure to SRIs

For each case patient we identified prescriptions for one of citalopram, escitalopram, fluvoxamine, paroxetine, and sertraline in the 90 days preceding the index date. We excluded fluoxetine because of the small number of cases exposed to this drug (n = 17).

### Statistical Analysis

We used standardized differences to compare baseline characteristics of cases and controls. Standardized differences of less than 0.1 indicate good balance between cases and controls for a given covariate (Austin et al., 2007).

We quantified the association between SRIs and cardiac toxicity in methadone-treated patients using two approaches. First, we used conditional logistic regression to estimate the odds ratio and 95% confidence intervals for the association between sudden cardiac death or ventricular dysrhythmia and receipt of a prescription for an SRI anticipated to increase the risk of these outcomes through either a pharmacokinetic (paroxetine, fluvoxamine, sertraline) or pharmacodynamic (citalopram and escitalopram) interaction with methadone. Patients not treated with an SRI served as the reference group. We adjusted all models for baseline variables with a standardized difference exceeding 0.1. Next, we constructed *p*-value functions to graphically convey the strength and precision of the relationship between SRIs and cardiac events among methadone-treated patients (Infanger and Schmidt-Trucksäss, 2019; Rothman et al., 2021). Because *p*-value functions display point estimates, one-sided and two-sided confidence limits at any level, and one-sided and two-sided *p* values for any null and non-null value in a single graph, they are more informative than single *p*-values or confidence intervals when presenting study findings (Infanger and Schmidt-Trucksäss, 2019; Rothman et al., 2021). Moreover, *p*–value functions provide an estimate of the counter-null value—the point estimate supported by the same amount of evidence as the null value of no effect—thereby discouraging dichotomization of results as “significant” or “non-significant” when drawing inferences (Greenland, 2017; Laber and Shedden, 2017; Infanger and Schmidt-Trucksäss, 2019; Rothman et al., 2021). Analyses were performed using SAS Enterprise Guide 7.1 (SAS Institute, Cary, North Carolina) and R Studio.

## Results

During the 21-year study period, we identified 960,933 patients who died of sudden cardiac death or were hospitalized with ventricular dysrhythmia. After exclusions, 670 of these individuals had been prescribed methadone within 1 day of death or hospitalization. Of the 670 patients, 626 (93.4%) were matched to at least one control. Overall, baseline characteristics of cases and controls were well balanced, with mean ages of 46.0 years (standard deviation ±11.6) and 45.3 years (standard deviation ±11.3), respectively (Table 1). As expected, case patients exhibited greater co-morbidity, received more prescription drugs in the preceding year, and had more visits with a cardiologist in the preceding year (Table 1).

**TABLE 1:** Characteristics of cases and controls.

Variable	Cases (n = 626)	Controls (n = 2,299)	Standardized difference^a^
Age (median, IQR)	47 (37–55)	46 (36–54)	0.06
18–34	16 (2.6%)	63 (2.7%)	0.01
35–44	113 (18.1%)	441 (19.2%)	0.03
45–64	141 (22.5%)	534 (23.2%)	0.02
65–74	198 (31.6%)	739 (32.1%)	0.01
75+	158 (25.2%)	522 (22.7%)	0.06
Female, No. (%)	227 (36.3%)	863 (37.5%)	0.03
Cardiologist visits in preceding year (median, IQR)	0 (0–1)	0 (0–1)	0.19
Charlson Co-morbidity Index, No. (%)			
No hospitalization	324 (51.8%)	1,266 (55.1%)	0.07
0	127 (20.3%)	523 (22.7%)	0.06
1	83 (13.3%)	267 (11.6%)	0.05
2 +	92 (14.7%)	243 (10.6%)	0.12
History of congestive heart failure, No. (%)	38 (6.1%)	80 (3.5%)	0.12
History of angina, No. (%)	17 (2.7%)	36 (1.6%)	0.08
History of acute myocardial infarction, No. (%)	34 (5.4%)	89 (3.9%)	0.07
History of hypertension, No. (%)	193 (30.8%)	616 (26.8%)	0.09
History of chronic kidney disease (3 years), No. (%)	16 (2.6%)	41 (1.8%)	0.05
Diabetes, No. (%)	112 (17.9%)	360 (15.7%)	0.06
Atherosclerotic disease, No. (%)	33 (5.3%)	90 (3.9%)	0.06
Stroke, No. (%)	12 (1.9%)	30 (1.3%)	0.05
Cardiomyopathy, No. (%)	6 (1.0%)	10 (0.4%)	0.06
Alcohol use disorder (3 years), No. (%)	54 (8.6%)	145 (6.3%)	0.09
Chronic liver disease (3 years), No. (%)	36 (5.8%)	89 (3.9%)	0.09
Residence in a long-term care facility, No. (%)	≤5^b^	≤5^b^	0.05
Number of prescription drugs in previous year, (median, IQR)	11 (7–16)	11 (6–15)	0.09
Medication use in preceding 120 days, No. (%)			
Non-potassium sparing diuretics	102 (16.3%)	282 (12.3%)	0.12
Potassium sparing diuretics^**b**^	6 (1.0%)	12 (0.5%)	0.05
Beta adrenergic receptor antagonists	58 (9.3%)	166 (7.2%)	0.07
ACE inhibitors	84 (13.4%)	258 (11.2%)	0.07
Angiotensin II receptor antagonists	21 (3.4%)	85 (3.7%)	0.02
Spironolactone	27 (4.3%)	54 (2.3%)	0.11
Potassium supplements	≤5^b^	≤5^b^	0.04
Direct renin inhibitors	0 (0%)	0 (0%)	0
Calcium channel blockers	61 (9.7%)	184 (8.0%)	0.06
Digoxin	≤5^b^	≤5^b^	0.01
Antiarrhythmic drugs	0 (0%)	≤5^b^	0.03
Nitrates	17 (2.7%)	38 (1.7%)	0.07
Anticoagulants	20 (3.2%)	46 (2.0%)	0.08
Antiplatelet drugs	16 (2.6%)	37 (1.6%)	0.07
Aspirin	190 (30.4%)	752 (32.7%)	0.05
Statins	68 (10.9%)	217 (9.4%)	0.05
Fibrates	≤5^b^	≤5^b^	0.01
Oral hypoglycemics	46 (7.3%)	158 (6.9%)	0.02
Insulin	37 (5.9%)	110 (4.8%)	0.05
Antipsychotic agents	170 (27.2%)	631 (27.4%)	0.01
Non-SRI antidepressants	200 (31.9%)	713 (31.0%)	0.02
Tricyclic antidepressants	88 (14.1%)	321 (14.0%)	0.00
Prokinetics	19 (3.0%)	78 (3.4%)	0.02
Opioids	176 (28.1%)	659 (28.7%)	0.01
Sedative-hypnotics	228 (36.4%)	866 (37.7%)	0.03
Cholinesterase inhibitors	≤5^b^	≤5^b^	0.02
Procedures in preceding 5 years No. (%)			
Coronary artery bypass graft	≤5^b^	10 (0.4%)	0.01
Angiography	36 (5.8%)	93 (4.0%)	0.08
Percutaneous transluminal coronary angioplasty	18 (2.9%)	39 (1.7%)	0.08
Permanent pacemaker insertion	0 (0.0%)	≤5^b^	0.05
Valve surgery	≤5^b^	15 (0.7%)	0.02
Carotid endartectomy	0 (0.0%)	0 (0.0%)	0.00
Echocardiography	209 (33.4%)	665 (28.9%)	0.10
Electrocardiography	540 (86.3%)	1967 (85.6%)	0.02
Holter monitor	54 (8.6%)	164 (7.1%)	0.06
Nuclear medicine stress test	32 (5.1%)	74 (3.2%)	0.09
Carotid Doppler ultrasonography	27 (4.3%)	73 (3.2%)	0.06
Income Quintile, No. (%)			
1 (lowest)	304 (48.6%)	1,105 (48.1%)	0.01
2	146 (23.3%)	549 (23.9%)	0.01
3	81 (12.9%)	294 (12.8%)	0
4	58 (9.3%)	229 (10.0%)	0.02
5	37 (5.9%)	122 (5.3%)	0.03

a Difference between cases and controls divided by standard deviation.

b Non-spironolactone potassium-sparing diuretics; prevalence not reported because of small cell size.

**FIGURE 1:**
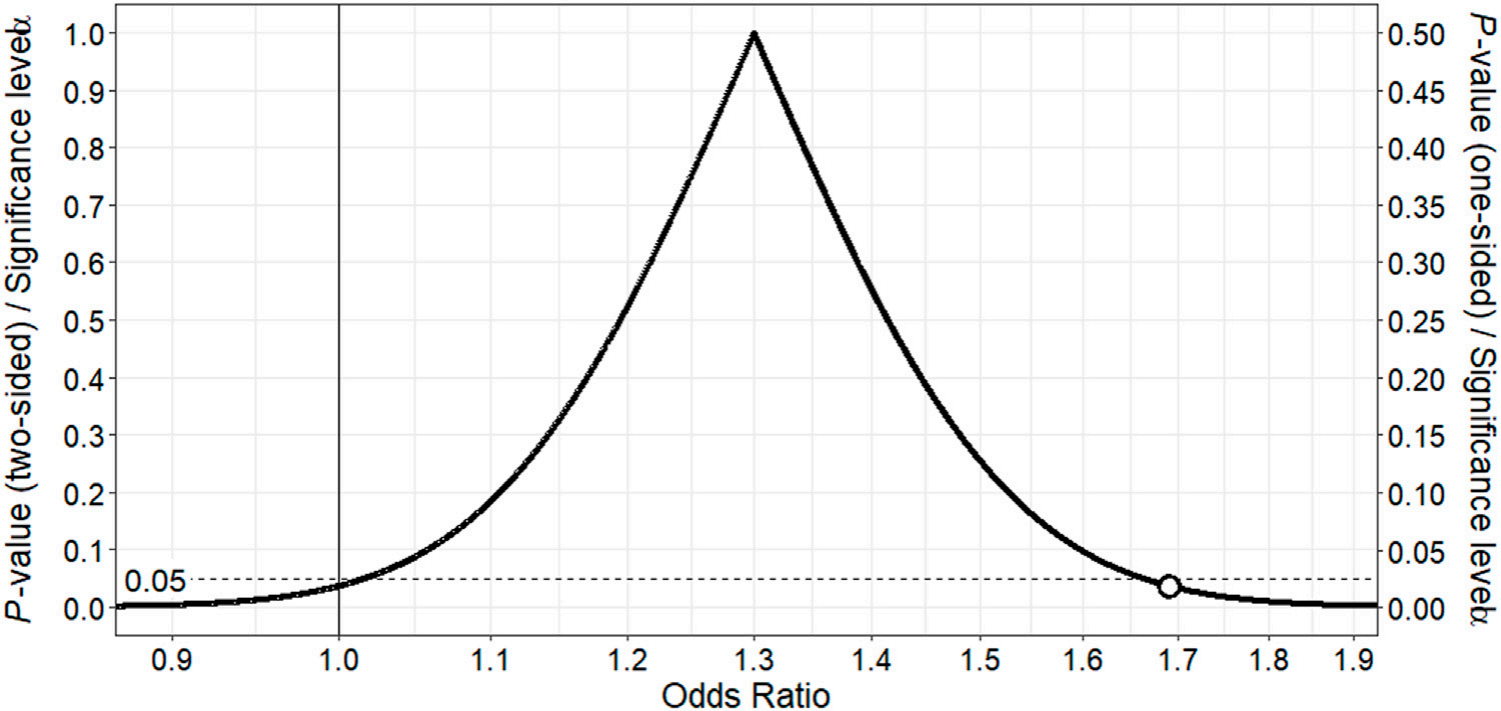
*p*-value function for odds ratio for association between fluvoxamine, paroxetine or sertraline and ventricular dysrhythmia or sudden death in methadone-treated patients. The point estimate of 1.30 corresponds to the peak of the *p*-value function. The vertical continuous line denotes the null value for the odds ratio, and the white point the counter-null value of 1.69, which is the effect size supported by the same amount of evidence as the null value.

Following multivariable adjustment, we found that use of sertraline, fluvoxamine or paroxetine (adjusted OR 1.30; 95% CI 0.90–1.86) was associated with a slightly increased risk of cardiac toxicity during methadone therapy (Table 2). The point estimate, representing the value most compatible with the observed data, is displayed at the peak of the corresponding *p*-value function (Figure 1). Importantly, the point estimate and a considerable portion of the range of effect values consistent with the data exceed 1, supporting an imprecise yet slightly higher risk of cardiac toxicity with these SRIs among methadone-treated patients relative to patients not treated with SRIs. Moreover, the counter-null value is 1.69, demonstrating that a 69% increase in the risk of cardiac toxicity is supported by the same amount of evidence as an odds ratio of 1.0.

**TABLE 2:** Association between sudden death or ventricular dysrhythmia and recent serotonin reuptake inhibitor use.

Serotonin Reuptake Inhibitor (SRI) Exposure in Preceding 90 days^a^	Patients		Odds ratio (95%confidence Interval)	Adjusted odds ratio† (95% confidence Interval)
	Cases (n = 626)	Controls (n = 2,299)		
	No. (%)	No. (%)		
Paroxetine/fluvoxamine/sertraline	44 (7.0%)	132 (5.7%)	1.30 (0.91–1.86)	1.30 (0.90–1.86)
Citalopram/escitalopram	93 (14.9%)	285 (12.4%)	1.24 (0.96–1.60)	1.26 (0.97–1.63)
No SRI	489 (78.1%)	1,882 (81.9%)	1.00	1.00

a Reference group: no SRI, use.

† Adjusted for congestive heart failure, spironolactone, non-potassium sparing diuretics, number of cardiologist visits and drug claims in preceding year, echocardiography in preceding 5 years.

**FIGURE 2:**
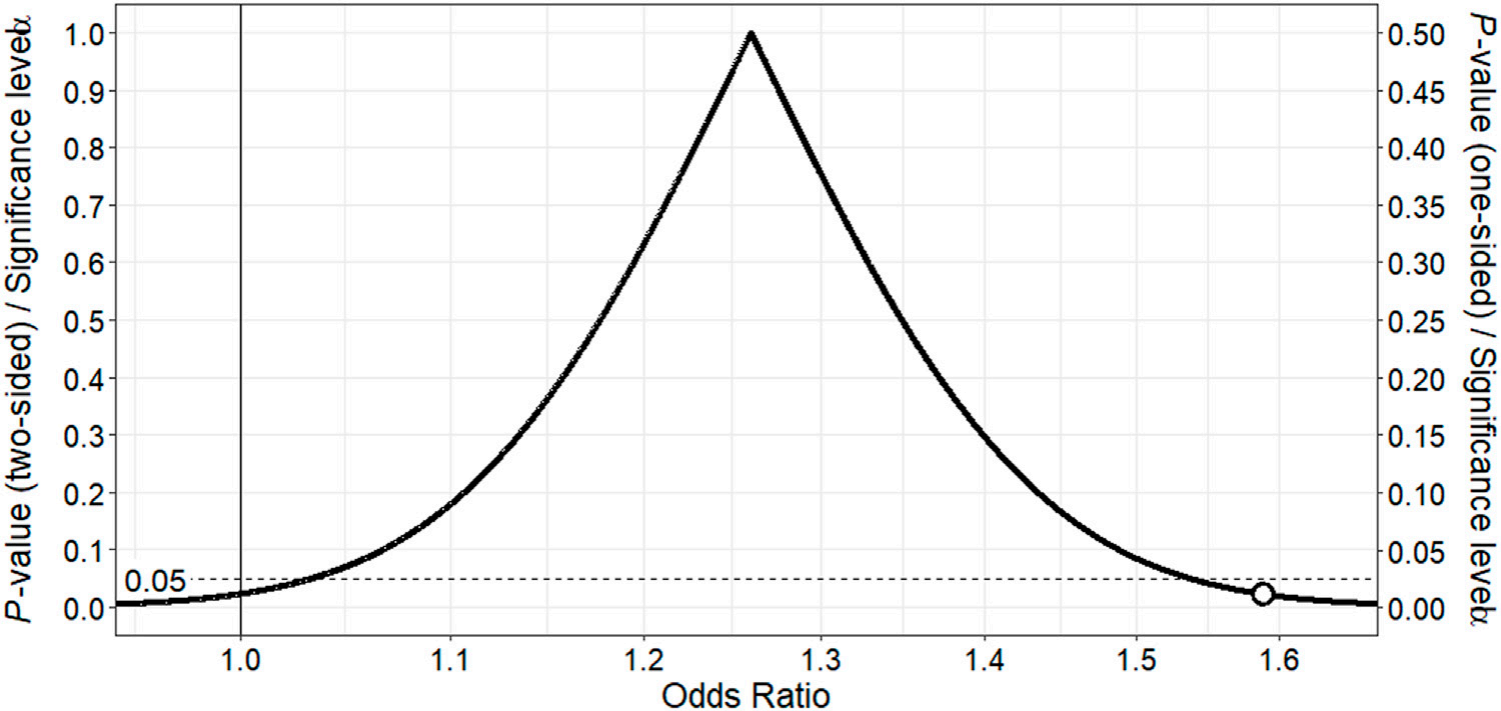
*p*-value function for odds ratio for association between citalopram or escitalopram and ventricular dysrhythmia or sudden death in methadone-treated patients.The point estimate of 1.26 corresponds to the peak of the *p*-value function. The vertical continuous line denotes the null value for the odds ratio, and the white point the counter-null value of 1.59, which is the effect size supported by the same amount of evidence as the null value.

Similarly, use of citalopram or escitalopram therapy was associated with a modestly higher risk of cardiac events (adjusted OR 1.26; 95% CI 0.97–1.63) relative to no SRI therapy (Table 2). The point estimate and most of the corresponding *p*-value function lie above 1, providing support for a slightly higher risk of cardiac toxicity with these SRIs in methadone-treated patients relative to no SRI therapy (Figure 2). The counter-null value is 1.59, demonstrating that a 59% increased risk in cardiac toxicity is supported by the same amount of evidence as a null finding of no risk.

## Interpretation

In this population-based study, we found that use of SRIs known to increase levels of (S)-methadone or prolong the QT interval were associated with a slight increase in the risk of dysrhythmias or sudden cardiac death in methadone-treated patients, an assertion supported by the individual point estimates and shapes of the corresponding *p*-value functions. Although the magnitude of the effect size is small, our findings support the existence of a potentially life-threatening drug interaction between methadone and certain SRIs in clinical practice.

Our findings build upon earlier research exploring interactions between SRIs and methadone. Specifically, past studies have found that fluvoxamine and paroxetine increase concentrations of (S)-methadone (Eap et al., 1997; Begré et al., 2002), and that this enantiomer is 3.5-times more potent than (R)-methadone in blocking the voltage-gated potassium channel of the human ether-a-*go-go* related gene (hERG) (Eap et al., 2007). Similarly, prior research demonstrating that sertraline inhibits CYP2B6 and that individuals with the slow metabolizer phenotype of CYP2B6 have higher (S)-methadone concentrations and longer QT intervals than individuals with normal CYP2B6 activity supports the notion of a clinically important interaction between methadone and sertraline (Hamilton et al., 2000). Because the QT interval has been found to increase by 19.2 s for every 1,000 ng/ml increase in (S)-methadone concentrations (Csajka et al., 2016), accumulation of this enantiomer following the co-administration of sertraline, fluvoxamine or paroxetine provides a reasonable mechanistic basis for the increased risk of cardiac toxicity with combined use. The finding of a higher risk of methadone-related cardiac toxicity with citalopram and escitalopram aligns with the known QT prolonging effects of these drugs (Castro et al., 2013; Beach et al., 2014). While this effect is likely of minimal significance in patients with no other risk factors for dysrhythmias, it may contribute to life-threatening QT interval prolongation in patients receiving concurrent therapy with proarrhythmic drugs such as methadone. Moreover, the combination of methadone and citalopram was invariably fatal in an exploratory study of drug combinations associated with opioid deaths, lending additional support to the notion of an important pharmacodynamic interaction between these drugs (Saad et al., 2018).

Our findings have important implications for public health. Methadone remains a cornerstone of therapy for the management of opioid use disorder, with the World Health Organization classifying it as an essential medication in 2005 (Herget, 2005). However, methadone-related QT interval prolongation and ventricular dysrhythmia are important contributors to methadone-related morbidity and mortality. Importantly, a community-based study of 22 cases of methadone-related sudden cardiac death at therapeutic doses identified an anatomical cardiac cause in only 23% of cases, with no clear cause identified for the remaining patients (Chugh et al., 2008). In contrast, a cardiac cause could be identified for 60% of non-methadone-related cases of sudden cardiac death. Although the overall risk of torsades de pointes is small and associated with multiple risk factors, our findings highlight an underappreciated drug interaction between methadone and commonly prescribed SRIs as a potential component cause in the occurrence of methadone-related cardiac toxicity, particularly among patients with no pre-existing cardiac risk factors for dysrhythmia. In light of our findings and past research, clinicians should follow standard methadone monitoring practices to mitigate the risk combined methadone-SRI therapy, including identification and management of risk factors for ventricular dysrhythmias, pre-treatment and follow-up electrocardiographic monitoring, and if clinically appropriate, selection of an antidepressant that does not interact with methadone.

Our study has some limitations. First, we used administrative data, and had no access to serum electrolytes, electrocardiograms, treatment adherence, and use of non-prescribed medications. Although we used validated codes for our outcomes, misclassification is possible. However, these limitations apply equally to all SRIs. Second, our study population comprised individuals eligible for public drug coverage in Ontario, which accounts for 70% of all methadone-treated patients in the province. Consequently, our findings may not be generalizable to all methadone-treated patients. Third, we were unable to reliably determine methadone dose. However, a dose-response relationship for methadone-related cardiotoxicity has not been clearly established, with cardiac effects documented at therapeutic doses (Chugh et al., 2008; Roy et al., 2012; Isbister et al., 2017). Fourth, some imbalance in baseline characteristics was apparent between cases and controls despite matching on a disease risk index. However, this is expected in case-control studies when cases are defined by an adverse outcome, and our analysis was adjusted for imbalanced variables. Finally, as with all observational studies, confounding due to unmeasured variables is a potential source of bias.

In conclusion, we found that SRIs expected to increase concentrations of the cardiotoxic (S)-methadone enantiomer or prolong the QT interval were associated with ventricular dysrhythmia and sudden cardiac death in patients receiving methadone. When combined therapy is required, the risks of a drug interaction can be minimized through careful patient selection that considers additional risk factors for QT prolongation and increased patient monitoring.

## Data Availability

The data analyzed in this study is subject to the following licenses/restrictions: The data set from this study is held securely in coded form at ICES. While data sharing agreements prohibit ICES from making the data set publicly available, access may be granted to those who meet prespecified criteria for confidential access (available at www.ices.on.ca/DAS). Requests to access these datasets should be directed to; www.ices.on.ca/DAS.
