# Supplementary material for: The Risk of Ventricular Dysrhythmia or Sudden Death in Patients Receiving Serotonin Reuptake Inhibitors With Methadone: A Population-Based Study
Source: Front Pharmacol. 2022 Apr 20;13:861953. doi: 10.3389/fphar.2022.861953 (PMC9065276; doi:10.3389/fphar.2022.861953)
Supplement: Supplementary file 1 [file DataSheet1.docx]

**Supplemental Table 1: ICD9 and ICD10 Codes for Cardiac Arrest and Ventricular Dysrhythmias**

| **ICD9 Code** | **Description** | **ICD10 Code** | **Description** |
| --- | --- | --- | --- |
| 427.5 | Cardiac arrest | I46 | Cardiac arrest |
|  |  | I47.0 | Reentry ventricular arrhythmia |
| 427.1 | Paroxysmal ventricular tachycardia | I47.2 | Ventircular tachycardia |
| 427.4 | Ventricular fibrillation and flutter | I49.0 | Ventricular fibrillation and flutter |
| 427.8 | Arrhythmia, other but not specified | I49.8 | Other specified cardiac arrhythmias |
| 427.9 | Arrhythmia (cardiac), NOS | I49.9 | Cardiac arrhythmia, unspecified |

**Supplemental Appendix – Covariates Included in Disease Risk Score**

Medication use in 120 days preceding index date

Non-potassium sparing diuretics

Potassium sparing diuretics

Beta-adrenergic receptor antagonists

Potassium supplements

Non-steroidal anti-inflammatory drugs

Angiotensin converting enzyme inhibitors

Angiotensin receptor blockers

Spironolactone

Calcium channel blockers

Digoxin

Antiarrhythmic drugs

Nitrates

Anticoagulants

Aspirin and other antiplatelet drugs

Statins

Fibrates

Oral hypoglycemic

Insulin

Antipsychotics

Antidepressants

Tricyclic antidepressants

Prokinetics

Opiates

Sedative hypnotics

Cholinesterase inhibitors

Comorbidities

Diabetes

Hypertension

Congestive heart failure

Chronic kidney disease

Chronic liver disease

Atherosclerotic disease

Myocardial infarction

Angina

Stroke/transient ischemic attack

Cardiomyopathy

Alcohol use disorder

Procedures in past 5 years:

Angiography

Carotid Doppler ultrasonography

Carotid endartectomy

Coronary artery bypass graft

Echocardiography

Electrocardiography

Holter monitor

Nuclear medicine stress test

Percutaneous transluminal coronary angioplasty

Permanent pacemaker insertion

Valve surgery

Number of cardiologist visits in past year

Number of prescription drugs in past year

Socioeconomic status (income quintiles)

Charlson co-morbidity index

Living in long-term care facility

Age

Sex
